# Reduced astrocytic reactivity in human brains and midbrain organoids with *PRKN* mutations

**DOI:** 10.1038/s41531-020-00137-8

**Published:** 2020-11-13

**Authors:** Masayoshi Kano, Masashi Takanashi, Genko Oyama, Asako Yoritaka, Taku Hatano, Kahori Shiba-Fukushima, Makiko Nagai, Kazutoshi Nishiyama, Kazuko Hasegawa, Tsuyoshi Inoshita, Kei-ichi Ishikawa, Wado Akamatsu, Yuzuru Imai, Silvia Bolognin, Jens Christian Schwamborn, Nobutaka Hattori

**Affiliations:** 1 Department of Neurology, Juntendo University Graduate School of Medicine, 2-1-1 Hongo, Bunkyo, Tokyo 113-8421 Japan; 2 Department of Neurology, Juntendo Koshigaya Hospital, 560 Fukuroyama, Koshigaya, Saitama 343-0032 Japan; 3 Department of Treatment and Research in Multiple Sclerosis and Neuro-intractable Disease, Juntendo University Graduate School of Medicine, 2-1-1 Hongo, Bunkyo, Tokyo 113-8421 Japan; 4 Department of Neurology, Kitasato University School of Medicine, 1-15-1 Kitasato, Minami-ku, Sagamihara, Kanagawa 252-0737 Japan; 5 Department of Neurology, National Hospital Organization Sagamihara National Hospital, 18-1 Sakuradai, Minami-ku, Sagamihara, Kanagawa 252-0392 Japan; 6 Department of Neurodegenerative and Demented Disorders, Juntendo University Graduate School of Medicine, 2-1-1 Hongo, Bunkyo, Tokyo 113-8421 Japan; 7 Center for Genomic and Regeneration Medicine, Juntendo University Graduate School of Medicine, 2-1-1 Hongo, Bunkyo, Tokyo 113-8421 Japan; 8 Department of Research for Parkinson’s Disease, Juntendo University Graduate School of Medicine, 2-1-1 Hongo, Bunkyo, Tokyo 113-8421 Japan; 9 Luxembourg Centre for Systems Biomedicine (LCSB), University of Luxembourg, 6 Avenue du Swing, L-4367 Belvaux, Luxembourg; 10 Braingineering Technologies SARL, 9 Avenue des Hauts-Forneaux, L-4362 Esch-sur-Alzette, Luxembourg

**Keywords:** Cellular neuroscience, Epigenetics, Parkinson's disease

## Abstract

Parkin (encoded by *PRKN*) is a ubiquitin ligase that plays an important role in cellular mitochondrial quality control. Mutations in *PRKN* cause selective dopaminergic cell loss in the substantia nigra and are presumed to induce a decrease in mitochondrial function caused by the defective clearance of mitochondria. Several studies have demonstrated that parkin dysfunction causes mitochondrial injury and astrocytic dysfunction. Using immunohistochemical methods, we analyzed astrocytic changes in human brains from individuals with *PRKN* mutations. Few glial fibrillary acidic protein- and vimentin-positive astrocytes were observed in the substantia nigra in *PRKN*-mutated subjects compared with subjects with idiopathic Parkinson’s disease. We also differentiated patient-specific induced pluripotent stem cells into midbrain organoids and confirmed decreased numbers of glial fibrillary acidic protein-positive astrocytes in *PRKN*-mutated organoids compared with age- and sex-matched controls. Our study reveals *PRKN*-mutation-induced astrocytic alteration and suggests the possibility of an astrocyte-related non-autonomous cell death mechanism for dopaminergic neurons in brains of *PRKN*-mutated patients.

## Introduction

Parkin RBR E3 ubiquitin-protein ligase *(PRKN)* is a causative gene for young-onset Parkinson’s disease (PD), and mutations in this gene are most frequent among young-onset PD patients^1^. The characteristic neuropathological feature of *PRKN* mutations is marked neuronal loss in the substantia nigra (SN) without Lewy pathology; however, there have been several reports of cases with α-synuclein-positive Lewy pathology in various regions^2–5^. In *PRKN*-mutated brains, neuronal loss is constant in the SN pars compacta (SNpc). When tissue damage and neuronal loss occur in the central nervous system (CNS), astrocytes usually react and change morphologically^6^. When considering astrocytic reactivity in individuals with *PRKN* mutations, several reports of *PRKN*-mutated pathologies have simply mentioned “gliosis” in the SNpc^7–13^, and only one report has described pathology without obvious astrocytosis in the SN^14^. In the human brain, astrocytes play many roles in the maintenance of the CNS environment, and they react and change morphologically in response to various triggers and regulators of environmental changes^6^. Astrocytic intermediate filaments (IFs) are general constituents of the cytoskeleton in astrocytes, and they act as a signaling platform that controls cell responses to various types of stress^15^. When astrocytes respond to CNS damage and change to reactive forms, the IF proteins glial fibrillary acidic protein (GFAP) and vimentin are upregulated^16^. These reactive phenomena are a pathological hallmark of reactive astrocytes^17^.

We investigated three autopsied patients with *PRKN* mutations who showed SNpc neuronal loss with or without α-synuclein pathologies, and with no astrocytic reactivity. Based on these pathological findings, we hypothesized that parkin dysfunction causes disturbances in astrocytic reactivity in *PRKN*-mutated brains. The present study evaluated astrocytic reactivity in the degenerated SN of *PRKN*-mutated patients; immunohistochemical methods were used to investigate the expression of IF proteins in this region. In addition, midbrain organoids (MOs) generated from induced pluripotent stem cells (iPSCs) reprogramed from patients’ fibroblasts also recapitulated this phenotype, and were used to investigate GFAP-positive astrocyte numbers.

## Results

### Clinical data of Control, idiopathic PD, and *PRKN*-mutated patients

Clinical data from all patients and controls are summarized in Table 1.

**Table 1:** Clinical data from patients and controls.

	Age (y/o)	Sex	Disease duration (y)	Mutation of *PRKN*
Parkin1	71	M	49	Exon 2/3 compound heterozygous deletion
Parkin2	74	M	43	Exon 2/3-4 compound heterozygous deletion
Parkin3 (PA)	72	F	10	Exon 2, 4 homozygous deletion
Parkin4	60	M	27	Exon 3 homozygous deletion
Parkin5 (PB)	50	M	22	Exon 6, 7 homozygous deletion
iPD1	76	F	31	
iPD2	72	F	22	
iPD3	76	M	20	
iPD4	72	M	6	
iPD5	76	M	12	
iPD6	73	M	12	
Cont1 (H1)	55	M		
Cont2 (H2)	68	F		
Cont3	79	M		
Cont4	54	M		
Cont5	73	M		
Cont6	81	M		

*y* years, *y/o* years old, *iPD* idiopathic Parkinson’s disease, *Cont* control.

### Neuropathology of *PRKN*-mutated patients

**Fig. 1:**
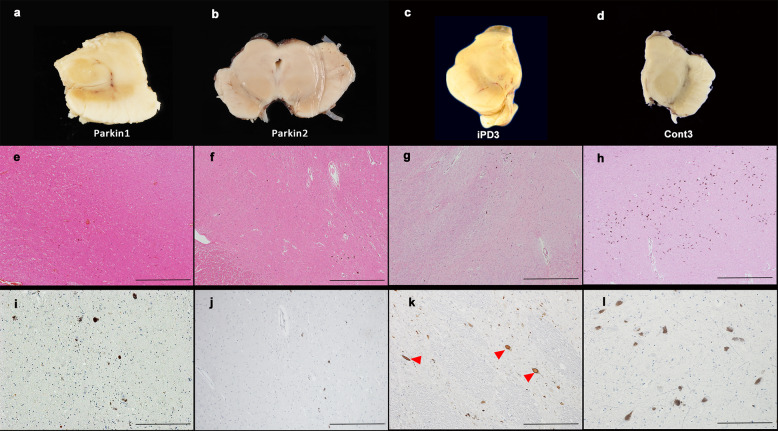
Neuropathology of the substantia nigra (SN) in parkin1, parkin2, idiopathic Parkinson’s disease 3 (iPD3), and normal control 3 (Cont3). SN pathologies in two *PRKN-*mutated patients, one idiopathic Parkinson’s disease (iPD) patient, and one normal control. Macroscopic photograph of the midbrains of each case (**a**–**d**). In hematoxylin and eosin-stained sections, marked neuronal loss was observed in the SN of parkin1 (**e**) and parkin2 (**f**), similar to that of iPD3 (**g**). The normal SN of Cont3 is also shown (**h**). No Lewy pathologies were observed in parkin1 (**i**) or parkin2 (**j**), whereas iPD3 showed α-synuclein-positive Lewy pathologies (**k**, red arrowheads). The normal SN of Cont3 is also shown (**l**). Scale bars represent 1 mm (**e**–**h**), and 200 µm (**i**–**l**).

The neuropathologies of two *PRKN*-mutated patients (parkin1 and 2), one idiopathic PD patient (iPD3), and one normal control (Cont3) are represented in Fig. 1. Macroscopically, the SN was normally pigmented (black appearance) in Cont3, but markedly depigmented in parkin1, parkin2, and iPD3 (Fig. 1a–d). With hematoxylin & eosin staining, the SN of both parkin1 (Fig. 1e) and parkin2 (Fig. 1f) showed severe neuronal loss (including of melanized neurons), similar to the SN of iPD3 (Fig. 1g), compared with Cont3 (Fig. 1h). However, no reactive astrocytes were observed in parkin1 or parkin2, unlike in iPD3. Furthermore, although α-synuclein-positive Lewy pathology was noted in iPD3 (Fig. 1k), no such pathology was observed in parkin1 (Fig. 1i) or parkin2 (Fig. 1j). The pathological data from parkin3 were described in a previous case report^5^ and are therefore not shown here.

**Fig. 2:**
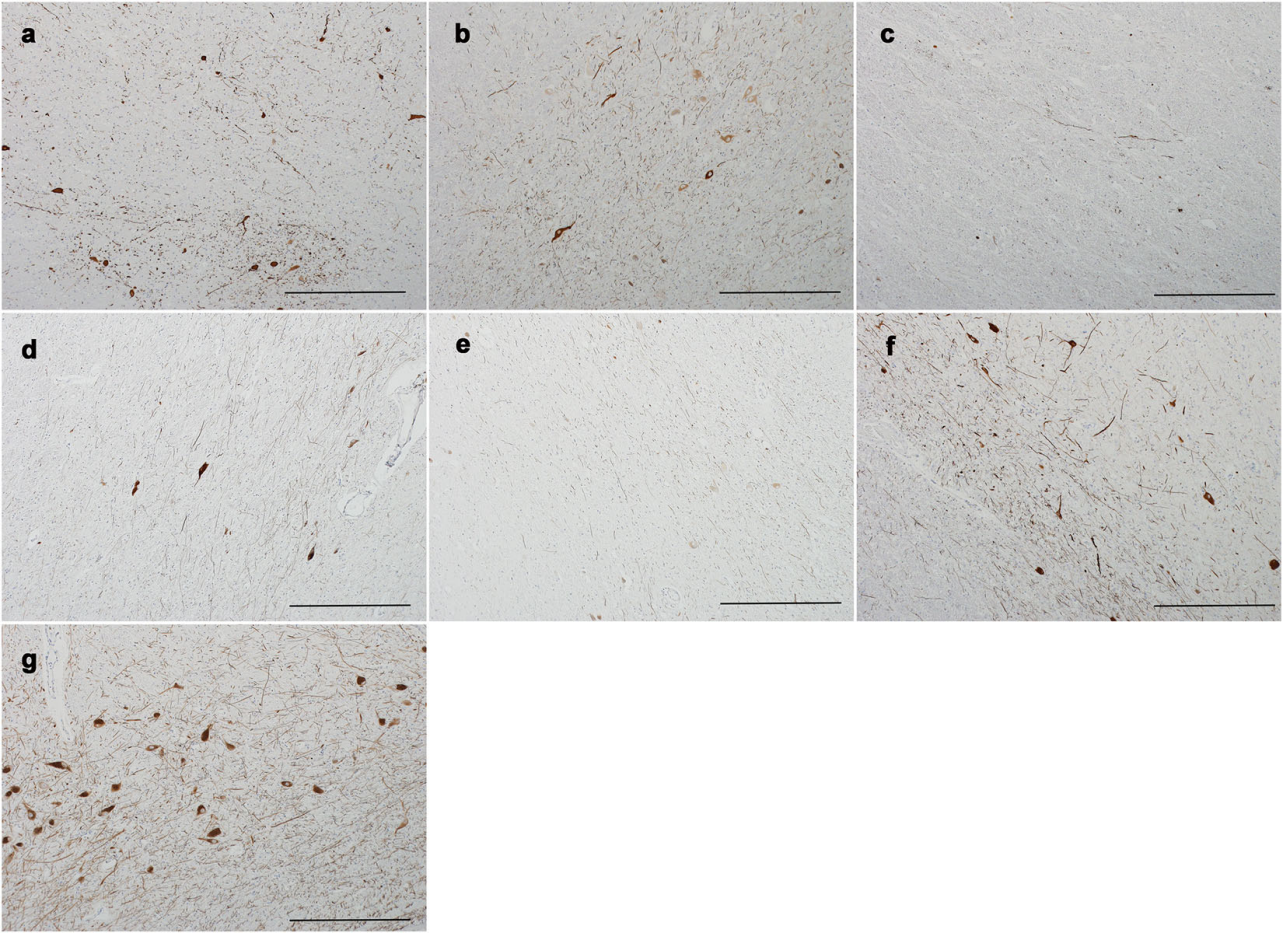
Immunohistochemistry of tyrosine hydroxylase (TH) in the substantia nigra pars compacta (SNpc) of *PRKN-*mutated patients, idiopathic Parkinson’s disease (iPD) patients, and a control. The TH-positive neurons in the SNpc were markedly lower in the *PRKN*-mutated and iPD patients compared with the control (Cont3), although the numbers varied among patients. The ventrolateral part of the SNpc is shown from parkin1 (**a**), parkin2 (**b**), parkin3 (**c**), iPD1 (**d**), iPD2 (**e**), iPD3 (**f**), and Cont3 (**g**). Scale bars represent 200 µm.

In tyrosine hydroxylase (TH) immunostaining, parkin1 (Fig. 2a), parkin2 (Fig. 2b), and parkin3 (Fig. 2c) had a marked but variable reduction in TH-positive neurons in the SN, similar to that observed in the iPD patients (Fig. 2d–f), compared with the control (Fig. 2g).

### Immunohistochemistry with astrocyte markers in *PRKN*-mutated brains

**Fig. 3:**
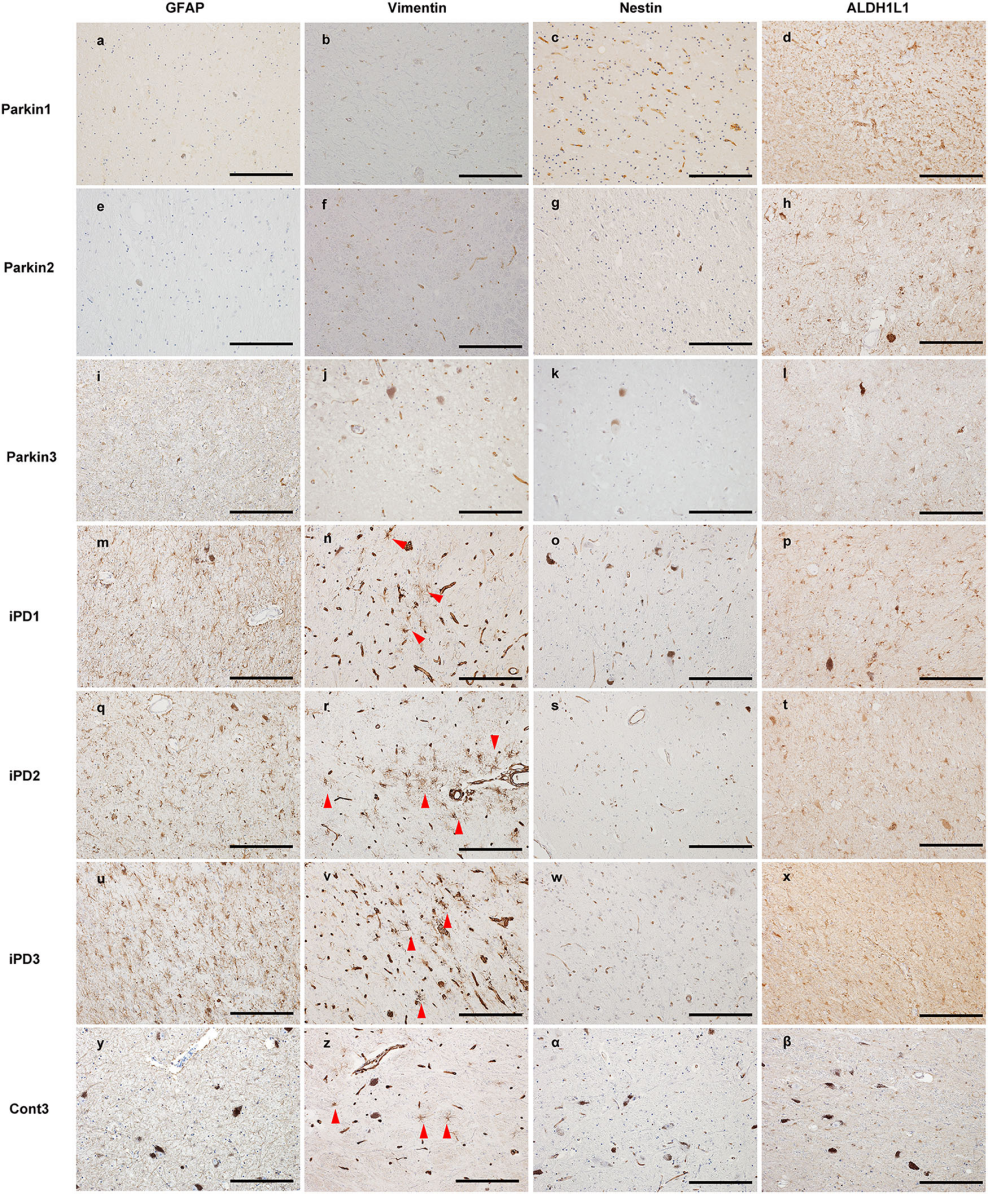
Immunohistochemical analysis using antibodies against astrocyte-related proteins. The substantia nigra (SNs) of *PRKN*-mutated patients were immunostained using anti-glial fibrillary acidic protein (GFAP; **a**), -vimentin (**b**), and -nestin (**c**) antibodies. The SN of the control (Cont3) and of idiopathic Parkinson’s disease (iPD) patients with long disease durations were also stained with anti-GFAP (**a**, **e**, **i**, **m**, **q**, **u**, **y**), -vimentin (**b**, **f**, **j**, **n**, **r**, **v**, **z**), and -nestin (**c**, **g**, **k**, **o**, **s**, **w**, **α**) antibodies. To identify stable astrocytes, immunohistochemistry against aldehyde dehydrogenase 1 family, member L1 (ALDH1L1) was also performed in the SN of all patients (**d**, **h**, **l**, **p**, **t**, **x**, **β**). Red arrowheads indicate positively stained astrocytes. All scale bars represent 200 µm.

We next examined astrocyte morphology in the SN of *PRKN*-mutated patients, iPD patients with disease duration of over 20 years, and normal control. We used immunostaining with antibodies against three molecular markers of astrocytic reactivity: GFAP, nestin, and vimentin. We also used antibodies against a marker of stable astrocytes: aldehyde dehydrogenase 1 family, member L1 (ALDH1L1). GFAP and vimentin are major constituents of IF proteins in mature astrocytes, and their expression markedly increases when astrocytes react to tissue injury in the CNS^16^. Therefore, enhanced GFAP expression is commonly considered a pathological hallmark of reactive astrocytes. Furthermore, under reactive conditions, nestin is re-expressed and vimentin is upregulated in astrocytes^18^. In the present study, the cell bodies and processes of non-reactive astrocytes were immunopositive for GFAP in the SN of the control. Furthermore, many GFAP-positive radial astrocytes were detected in the SN of iPD patients with a disease duration of more than 20 years. In contrast, no GFAP-positive reactive astrocytes were observed in the SN of parkin1 or parkin2, although there was marked neuronal loss. In the SN of parkin3, astrocytes were faintly stained, with thin shapes and radial processes (Fig. 3a, e, i, m, q, u, y). In the SN of control and iPD patients, there were some and many vimentin-positive astrocytes, respectively; however, no vimentin-positive astrocytes were observed in the SN of parkin1, parkin2, or parkin3 (Fig. 3b, f, j, n, r, v, z). Nestin was not detected in the SN of the control, iPD patients, or *PRKN*-mutated patients (Fig. 3c, g, k, o, s, w, α). Conversely, ALDH1L1 immunostaining revealed positively stained astrocytes in all cases (Fig. 3d, h, l, p, t, x, β). These immunohistochemical results for astrocyte markers suggested that (1) stable astrocytes (ALDH1L1-positive) existed in the SN of both iPD and *PRKN*-mutated patients; (2) even after a long disease duration (more than 20 years), GFAP and vimentin reactivity was detected in iPD patients; (3) GFAP reactivity in astrocytes was diminished in the SN of parkin1 and parkin2 (over 40 years of disease duration), but staining was faint in parkin3, who had a relatively short disease duration (10 years) and atypical Lewy pathologies; and (4) vimentin was not detected in the SN of any *PRKN*-mutated patients, regardless of disease duration.

### Immunohistochemistry and expression assays of astrocyte-related proteins in the cerebral cortex of *PRKN*-mutated patients, iPD patients, and controls

**Fig. 4:**
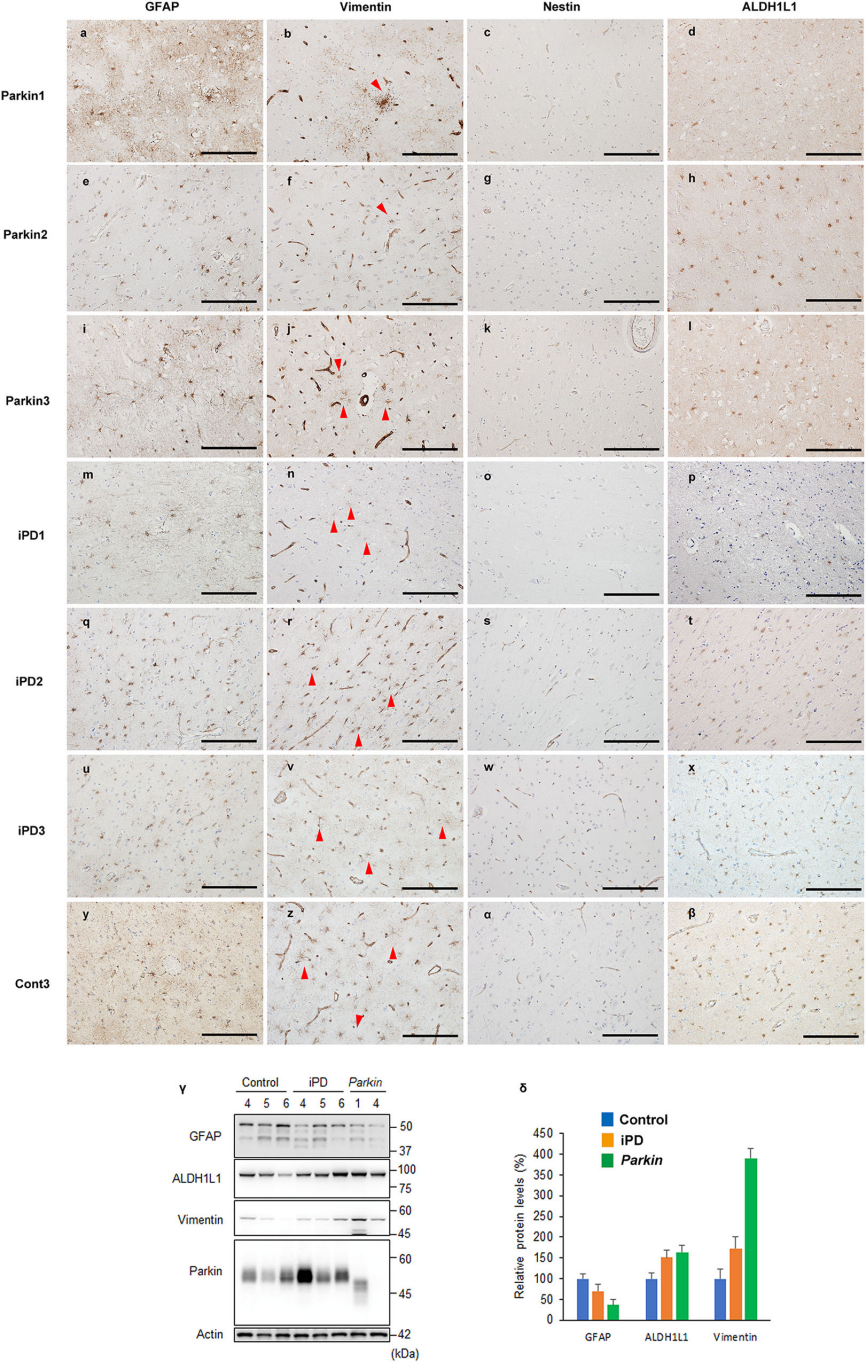
Immunohistochemistry and protein expression of astrocyte-related proteins in the frontal cortex of *PRKN*-mutated patients, idiopathic Parkinson’s disease (iPD) patients, and a control. Immunohistochemistry against glial fibrillary acidic protein (GFAP) (**a**, **e**, **i**, **m**, **q**, **u**, **y**), vimentin (**b**, **f**, **j**, **n**, **r**, **v**, **z**), nestin (**c**, **g**, **k**, **o**, **s**, **w**, **α**), and aldehyde dehydrogenase 1 family, member L1 (ALDH1L1) (**d**, **h**, **l**, **p**, **t**, **x**, **β**). Astrocytic GFAP, vimentin, and ALDH1L1 were detected in the frontal cortex of the control, idiopathic Parkinson’s disease (iPD) patients, and *PRKN*-mutated patients. Red arrowheads indicate positively stained astrocytes. Scale bars represent 200 µm (**a**–**β**). The western blot analysis from control, iPD, and *PRKN*-mutated frontal cortices was performed using GFAP, vimentin, ALDH1L1, and parkin antibodies (**γ**). Actin served as the loading control. A quantitative analysis of the relative protein levels of GFAP, vimentin, and ALDH1L1 revealed no statistically significant differences among the *PRKN*-mutated patients, iPD patients, and controls (**δ**).

To confirm whether GFAP and vimentin were decreased not only in the SN but also in other brain regions, we performed immunohistochemistry using astrocytic markers in the frontal cortex, which usually shows no neurodegenerative changes in patients with *PRKN* mutations. Nestin, which is expressed in the immature phase and re-expressed under acute reactive conditions, was not detected in these patients (Fig. 4c, g, k, o, s, w, α). In contrast, GFAP (Fig. 4a, e, i, m, q, u, y), vimentin (Fig. 4b, f, j, n, r, v, z), and ALDH1L1 (Fig. 4d, h, l, p, t, x, β) positivity were observed in the astrocytes of the three *PRKN*-mutated patients.

To biochemically evaluate the expression of astrocytic markers in the *PRKN*-mutated brains, we performed western blots for GFAP, vimentin, and ALDH1L1 using postmortem tissue from the frontal cortex (Fig. 4γ). Multiple bands representing the different GFAP isoforms were detected in the *PRKN*-mutated brains, as well as in the brains of iPD patients and controls^19^. Vimentin and ALDH1L1 bands were also expressed in all cases. We also performed a quantitative analysis of GFAP, vimentin, and ALDH1L1 from these western blot data (Fig. 4δ). Although the band intensities of each protein varied among the cases, there were no statistically significant differences in GFAP, vimentin, or ALDH1L1 expression in the frontal cortex. These results suggest that astrocytes are not markedly altered in the *PRKN*-mutated frontal cortex, in which neurodegeneration was not detected in the *PRKN*-mutated brains.

### GFAP immunoreactivity analysis in *PRKN*-mutated MOs

**Fig. 5:**
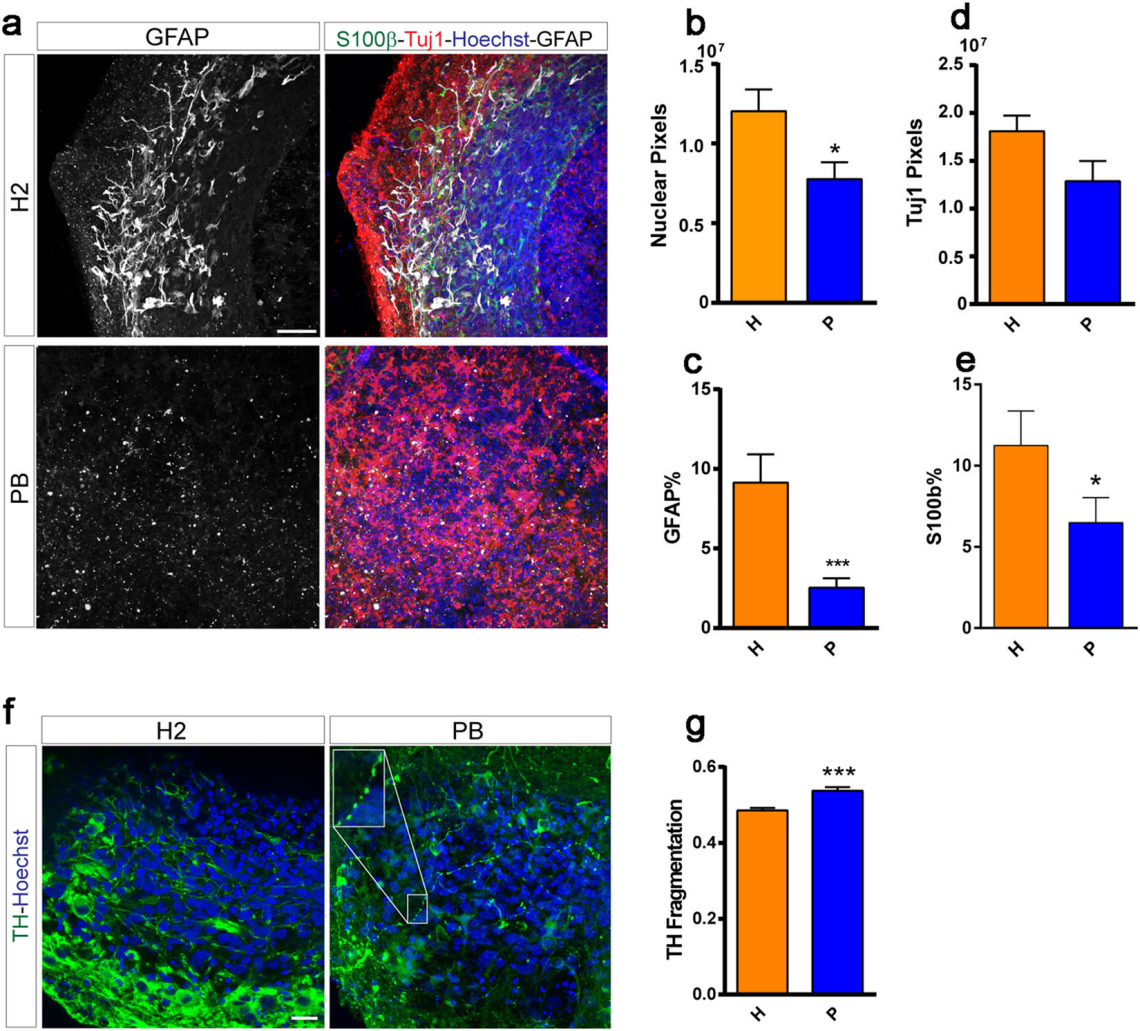
Immunofluorescence staining and image analysis of human midbrain organoids (MOs). Organoids carrying *PRKN* mutations showed decreased glial fibrillary acidic protein (GFAP) immunoreactivity and increased tyrosine hydroxylase (TH)-positive neuron fragmentation compared with controls. P represents human-derived MOs from parkin3 and parkin5; H represents data from two controls. Representative confocal images show GFAP, S100β, Hoechst, and Tuj1 staining in the H and P lines (**a**). The total number of Hoechst-positive pixels was quantified using Matlab algorithms (**b**). Automated quantification of the total amount of Tuj1-positive pixels (**d**), and of the percentage of GFAP- (**c**), and S100β-positive cells (**e**), was also carried out using Matlab algorithms. Representative confocal images showing TH and Hoechst staining in the H and P lines (**f**). Automated quantification of TH-positive neuron branching fragmentation as an indicator of degeneration (**g**). Statistical analysis was performed using Mann–Whitney tests (*p < 0.05, ***p < 0.001). Scale bars represent 50 µm in **a** and 20 µm in **f**. For each cell line, three organoids each from H1 and H2 and six organoids each from PA and PB were used for the analysis. At least three 50 µm sections were imaged for each organoid. We performed automatic quality control selection to remove any sections with a Hoechst pixel sum of below 5000. For **e**, a total of 9 and 15 sections were analyzed for each of H1/H2 and PA/PB, respectively. For **g**, a total of 10 (H1), 9 (H2), 17 (PA), and 15 (PB) sections were analyzed. An entire organoid section represents a data point in the graphs and was averaged with the other sections.

To further investigate the effects of *PRKN* mutations on astrocytes, we generated human MOs that were differentiated for 35 days (Fig. 5). Overall, the human MOs derived from *PRKN-*mutation carriers were smaller than those derived from age- and sex-matched controls; this finding was recapitulated by the significantly lower Hoechst-positive pixel counts (Fig. 5a, b). Expression levels of the neuronal marker Tuj1 were similar among the different cell lines (Fig. 5c). In contrast, the percentage of GFAP-positive cells in human MOs was significantly lower in *PRKN-*mutation carriers than in healthy individuals (Fig. 4d). The percentage of cells positively stained with S100β also tended to be lower in *PRKN-*mutation carriers (Fig. 5e). When analyzing TH-positive neurons, we observed increased fragmentation in the neuronal processes in human MOs from *PRKN*-mutated patients compared with controls (Fig. 5f, g). This is indicative of dopaminergic neuron degeneration. Overall, the patient-specific MOs recapitulated some of the key features that were observed in the postmortem brain samples. A description of the features extracted from the image analysis is shown in Table 2.

**Table 2:** Description of the features extracted from the image analysis.

Features	Description
GFAP percentage	% of GFAP-positive cells
Tuj1 pixels	Sum of Tuj1-positive pixels
TH fragmentation	Surface to volume ratio of TH mask
Nuclear pixels	Sum of Hoechst-positive pixels

## Discussion

In the present study, we revealed reduced reactivity of the astrocytic IF protein GFAP in the degenerated SN of *PRKN*-mutated patients, as well as in MOs generated from iPSCs reprogramed from *PRKN*-mutated patient fibroblasts.

When CNS injury occurs, astrocytes react to the pathological environment and change their morphology, exhibiting a hypertrophic cell body and processes^6^. In reactive astrocytes, genes encoding the IF proteins GFAP, vimentin, and nestin are overexpressed or re-expressed^18^. Under such conditions, IF proteins can be visualized in astrocytes using immunohistochemistry^6,20^. If the triggering insult is removed or decreases, these structural changes resolve over time^18^. However, the reactive profile of a number of upregulated astrocytic genes, including *GFAP*, can remain from several hours to days or even decades^21^. In our study, we were able to detect sufficient numbers of GFAP- and vimentin-positive reactive astrocytes in the SN of iPD patients with long disease durations. Of the *PRKN*-mutated patients, the two patients (parkin1 and parkin2) without α-synuclein-related pathologies and with long disease duration (over 40 years) had no GFAP-positive astrocytes. The patient (parkin3) with α-synuclein-related pathologies similar to those of iPD patients, and with a relatively short disease duration for an individual with a *PRKN* mutation (10 years), showed mild GFAP-positive pathology in the SN compared with the iPD patients. α-synuclein-related pathologies meant widespread α-synuclein-positive Lewy pathologies and neurodegeneration (neuronal loss and astrocytosis) in the SN, locus ceruleus, and vagal nucleus. We believe that parkin3 was an atypical *PRKN*-mutated case that co-existed with iPD, because this patient had late-onset PD (aged 61 years) and showed widespread α-synuclein-related pathologies, from non-CNS organs to the brainstem and limbic regions^5^. Vimentin-positive astrocytes were not present in the SN of any of the three *PRKN*-mutated patients. These findings suggest that the upregulation of GFAP and vimentin might be insufficient in the SN of *PRKN*-mutated brains, even when the neurodegenerative process is occurring. Regarding astrocytic pathologies in patients with *PRKN* mutations, only one report has described pathology without any obvious Lewy pathologies or astrocytosis in the SN, in a *PRKN*-mutated patient with a disease duration of 44 years^14^. Our data and those of this previous report suggest that the expression of GFAP and vimentin in astrocytes is not activated in the α-synuclein-negative neurodegeneration that is induced by *PRKN* mutations. They also suggest that GFAP might upregulate insufficiently to additional neurodegenerative processes even within short disease durations. These findings were not caused by decreased astrocytic numbers, because ALDH1L1-positive astrocytes were present in the SN of both iPD and *PRKN*-mutated patients. The decreased IF immunoreaction in the SN of *PRKN*-mutated patients may therefore indicate a failure to maintain astrocytic reactivity.

Moreover, we demonstrated a decrease in GFAP- and S100β-positive astrocytes in *PRKN*-mutated patient-specific human MOs for the first time. This result suggests a possibility that the failure of astrocytic IF reactivity that was observed in *PRKN*-mutated brains may have been caused by developmental abnormalities of astrocytic IFs in *PRKN*-mutated brains in these individuals.

The prominent feature of *PRKN*-mutated pathology is the selective loss of neurons in the SNpc^22^. Thus, the regional restriction of decreased GFAP and vimentin immunoreactivity observed in the present study may be related to the selective neuronal cell loss that occurs in the SNpc.

The role of astrocytes in brains with dysfunctional parkin remains unclear. Parkin is a ubiquitin E3 ligase that retains mitochondrial quality via the removal of damaged mitochondria by mitophagy^23–27^. A previous study reported increased levels of damaged astrocytic mitochondria in mice with *PRKN* exon 3 deletion compared with healthy controls^28^. Moreover, cell biology studies have demonstrated that both parkin dysfunction and reduced levels of parkin can impair astrocytic function^29,30^. Furthermore, GFAP expression levels are reportedly decreased in astrocytic cultures from *PRKN* knockout mice^31^. These previous experimental studies reported that astrocytic dysfunction is associated with decreased GFAP expression, which can be induced either in vitro or in animal models by parkin impairment or reduction, and by increasing levels of damaged mitochondria. In our human immunohistochemistry studies, the lack of IF reactivity in the SN suggests that astrocytic dysfunction is induced by parkin dysfunction.

Astrocytes can be classified into two subtypes: A1 and A2. A1 astrocytes are induced by inflammation and are “harmful”, whereas A2 astrocytes are induced by ischemia and are “helpful” for neurons^21^. It has been reported that mRNAs for GFAP and vimentin are upregulated in both A1 and A2 astrocytes^32^. Furthermore, in a previous study of mice carrying null mutations in the genes for both GFAP and vimentin, these mice (which lacked astrocytic cytoplasmic IFs) had attenuated reactive astrocytosis, impaired glial scar formation, more prominent synaptic loss after neurotrauma, and CNS tissue that was less resistant to mechanical stress. However, they also showed some positive outcomes, with better synaptic and axonal regeneration after traumatic injuries of the CNS^16^. These findings suggest that the failure of astrocytic IF reactivity has two aspects: “harmful” and “helpful”. However, it remains unclear whether the failure of astrocytic IF reactivity affects dopaminergic cell loss in the SN of subjects with *PRKN* mutations. Our pathological data showed marked but varied reductions in TH-positive neurons in the SN of *PRKN*-mutated patients, similar to what was observed in the iPD patients with or without astrocytic IF reactions. We were unable to detect any differences between *PRKN*-mutated and iPD patients in terms of the relationship between astrocytosis and dopaminergic cell loss in this study because our human sample numbers were small. However, a previous study reported that iPSC-derived astrocytes from a PD patient with an *LRRK2* G2019S mutation showed impaired autophagy and accelerated α-synuclein aggregation, which contributed to the degeneration of SN dopaminergic cells^33^. This evidence of an astrocytic contribution to dopaminergic cell death suggests a crucial role for non-autonomous neuronal cell death in PD.

In summary, we revealed that astrocytic alterations are induced by *PRKN* mutations. The main limitation of the present study was the relatively small number of human samples that were available. We were unable to obtain more brain tissue because *PRKN*-mutated PD patients are very uncommon and autopsies of these patients are extremely rare. As a result of this limitation, we were unable to elucidate the relationship between astrocytic alterations and dopaminergic cell loss. Nevertheless, we were able to reveal a failure of astrocytic IF reactivity in the *PRKN*-mutated SN using human brain specimens, and recapitulated astrocytic pathologies in human *PRKN*-mutated iPSC-derived MOs for the first time. Moreover, the present study highlighted the potential of patient-specific MOs for the in vitro modeling of PD. Further investigations focused on the relationship between neuronal loss and astrocytic dysfunction in the SN of *PRKN*-mutated human patients are therefore warranted.

In conclusion, parkin dysfunction affects astrocytic IF reactivity, which may be associated with neurodegeneration in *PRKN*-mutated brains.

## Methods

### Control, idiopathic PD, and *PRKN*-mutated patients

For neuropathological and immunohistochemical analyses, we used brain tissue from three autopsied *PRKN*-mutated patients (parkin1–3), three pathologically confirmed idiopathic PD patients (iPD1–3), and one control (Cont3: amyloidosis without CNS pathologies). Furthermore, frozen brain tissue from two *PRKN*-mutated patients (parkin1 and parkin4), three iPD patients (iPD4–6), and three controls (Cont4–6) was used to analyze the expression of astrocyte-related proteins in the frontal cortex. For the human MO analysis, iPSCs were derived from two *PRKN*-mutated patients (parkin3: PA; parkin5: PB) and two normal controls (Cont1: H1; Cont2: H2).

Parkin3 was the first reported case of nigral degeneration with Lewy pathology in an individual with a *PRKN* mutation^5^, and the iPSC phenotypes of this patient were described in our previous report as patient PA^34^. The iPSC phenotypes of parkin5 were described in our previous report as patient PB^34^. Neuropathological analyses of parkin4 and parkin5 were not available.

This study was approved by the ethics committee of the Juntendo University School of Medicine (approval numbers: 2019012, 2017032, 2012068) in accordance with the Code of Ethics of the World Medical Association (Declaration of Helsinki). Before participating, all patients gave full written informed consent for the experimental procedures using brain autopsy tissue and human iPSCs.

### Neuropathology

Neuropathological specimens were fixed with 15% formalin and embedded in paraffin. The SNs of parkin1–3, iPD1–3, and Cont3 were evaluated pathologically using hematoxylin and eosin staining, Klüver–Barrera staining, α-synuclein immunostaining (anti-phosphorylated α-synuclein mouse monoclonal antibody, CAT# 015-29151, FUJIFILM Wako, Chuo-Ku, Osaka, Japan; dilution 1:1000), and TH immunostaining (TH mouse monoclonal antibody, CAT# T2928m, Sigma-Aldrich, St. Louis, MO, USA; dilution 1:5000).

### Immunohistochemical analysis of astrocytes

We evaluated astrocytic reactivity in the SN and frontal cortex of parkin1–3, iPD1–3, and Cont3 using immunohistochemistry for three IF proteins: GFAP, nestin, and vimentin. These proteins are upregulated or re-expressed when astrocytes react and form IF networks^16,35^. The primary antibodies used were anti-nestin rabbit polyclonal antibody (CAT#18741, Imuno-Biological Laboratories (IBL), Fujioka, Gunma, Japan; dilution 1:200), anti-GFAP mouse monoclonal antibody (CAT#11051, IBL; dilution 1:200), and anti-vimentin mouse monoclonal antibody (CAT# GA63061-2J, Dako, Santa Clara, CA, USA; dilution 1:200). We also performed immunohistochemistry for stable astrocytes using anti-ALDH1L1 rabbit polyclonal antibody (CAT# 17390-1-AP, Proteintech, Rosemont, IL, USA; dilution 1:200).

### Western blot analysis of parkin and astrocyte-related proteins in human brain tissue

We performed expression assays of astrocyte-related proteins using postmortem autopsied frontal cortexes. The subjects included three controls with no pathological brain lesions (Cont3–5), three iPD patients (iPD4–6), and two patients with *PRKN* mutations (parkin1 and parkin4). The clinical data of the controls were not available.

Frozen human brain tissue containing the cerebral cortex was homogenized on ice in radioimmunoprecipitation assay buffer containing protease inhibitor cocktail (Nacalai Tesque, Nakagyo, Kyoto, Japan) before being centrifuged at 20,400×*g* for 10 min at 4 °C. Next, 10 µg of the supernatant, resolved in 3× Laemmli sample buffer, was subjected to western blotting with anti-GFAP mouse monoclonal antibody (CAT# G3893, Sigma-Aldrich, St. Louis, MO, USA; dilution 1:1000; clone G-A-5), anti-vimentin mouse monoclonal antibody (CAT# V6630, Sigma-Aldrich; dilution 1:200), anti-ALDH1L1 rabbit polyclonal antibody (CAT# 17390-1-AP, Proteintech, Rosemont, IL, USA; dilution 1:5000), anti-parkin mouse monoclonal antibody (CAT# 4211, Cell Signaling Technology, Danvers, MA, USA; dilution 1:1000; clone PRK8), and anti-actin mouse monoclonal antibody (CAT# MAB1501, Millipore, Burlington, MA, USA; dilution 1:10,000; clone C4). Western blot detection was performed with ECL prime solution (Cytiva, Shinjuku, Tokyo, Japan), and images of the blots were obtained using an Image Quant LAS 4000 mini (Cytiva).

The relative protein levels (normalized to actin levels) were quantitatively analyzed against normal controls and compared statistically using JMP software (version 11.0.0, Cary, NC, USA).

### iPSC cultures

We used iPSCs from two PD patients carrying *PRKN* mutations (PA and PB) and two healthy age-matched controls (H1 and H2) in this study. Detailed information about the iPSC clones is provided in our previous report^34^. Maintenance of the iPSCs was performed in E8 medium, on a Matrigel matrix. Cultures were split using Accutase, followed by overnight incubation with 5 µM Y-27632 (Merck Millipore, Burlington, MA, USA).

### Derivation of ventralized neuroepithelial stem cells

Ventralized neuroepithelial stem cells were generated from iPSCs. The iPSCs were detached using Accutase (Sigma-Aldrich) and collected in embryoid body (EB) medium, consisting of Knockout DMEM (Invitrogen, Carlsbad, CA, USA) with 20% KnockOut Serum Replacement (Invitrogen), 100 µM β-mercaptoethanol (Gibco, Waltham, MA, USA), 1% nonessential amino acids (Invitrogen), and 1% penicillin/streptomycin/glutamine (Invitrogen), freshly supplemented with 10 µM SB-431542 (SB; Ascent Scientific, Cambridge, Cambridgeshire, UK), 150 nM LDN-193189 (LDN; Sigma-Aldrich), 3 µM CHIR99021 (CHIR; Axon Medchem, Groningen, the Netherlands), 0.5 µM SAG (Merck, Branchburg, NJ, USA), and 5 µM ROCK inhibitor (Sigma-Aldrich). EBs were formed with 2000 iPSCs each using AggreWell 400 (STEMCELL Technologies, Vancouver, BC, Canada). After 24 h, EBs were harvested in EB medium without ROCK inhibitor and transferred to a non-treated tissue culture plate (Corning, Corning, NY, USA). On day 2, the medium was replaced with N2B27 (as described above) supplemented with 10 µM SB, 150 nM LDN, 3 µM CHIR, and 0.5 µM SAG. The medium was changed with the addition of 200 µM ascorbic acid (AA; Sigma-Aldrich) on days 3 and 4. On day 8, colonies with neuroepithelial outgrowth were collected, triturated with a 1000 mL pipette, and transferred in a 1:10 ratio to a 24-well plate (Corning). Cells were split three to four times while being kept in maintenance media (3 µM CHIR, 2.5 µM SB, 100 nM LDN, 0.5 µM SAG, and 200 µM AA).

### Generation of human MOs

To generate midbrain-specific organoids, 3000 cells/well were seeded in an ultra-low-attachment 96-well round-bottom plate and kept under maintenance conditions for 7 days. To begin patterning, LDN and SB were withdrawn. After 3 additional days, the CHIR concentration was reduced to 0.7 µM. On day 9 of differentiation, the medium was changed to neuronal maturation medium, including 200 µM AA, 10 µM DAPT, 500 µMdb cAMP, 10 ng/mL human brain-derived neurotrophic factor, 10 ng/mL human glial cell-derived neurotrophic factor, 1 ng/mL TGF-3β, and 2.5 ng/mL Activin A. The organoids were kept under static culture conditions for 35 days with media changes every 3 days.

### Immunofluorescence staining of human MOs

Human MOs were fixed overnight at 4 °C with 4% paraformaldehyde, washed three times with phosphate-buffered saline (PBS) for 15 min, and embedded in 3–4% low-melting-point agarose in PBS. The solid agarose block was sectioned at 50 µm using a vibratome (Leica VT1000s), and sections were then blocked for 90 min at room temperature on a shaker with 0.5% Triton X-100, 0.1% sodium azide, 0.1% sodium citrate, 2% bovine serum albumin, and 5% normal goat or donkey serum in PBS. Primary antibodies were diluted in the same solution but with only 0.1% Triton X-100 and sections were incubated for 48 h at 4 °C. The following primary antibodies were used: anti-Tuj1 rabbit polyclonal antibody (Optim AB Eurogentec, Liège, Belgium; dilution 1:600), anti-GFAP chicken polyclonal antibody (CAT#AB5541, Millipore; dilution 1:1000), anti-S100β mouse monoclonal antibody (CAT# AMAB91038, Sigma-Aldrich; dilution 1:1000), and anti-tyrosine hydroxylase (TH) rabbit polyclonal antibody (CAT# 657012, Santa Cruz Biotechnology, Dallas, TX, USA; dilution 1:1000).

Sections were then washed three times with PBS, incubated for 2 h at room temperature with the secondary antibodies in 0.05% Tween-20 in PBS, washed with 0.05% Tween-20 in PBS and Milli-Q water, and mounted in Fluoromount-G mounting medium (Southern Biotech, Birmingham, AL, USA). The organoids were imaged using an Operetta confocal microscope (PerkinElmer, Waltham, MA, USA).

### Image analysis of human MOs

Immunofluorescent three-dimensional images of human MOs were analyzed using Matlab (v2017b, MathWorks, Natick, MA, USA). The in-house-developed image analysis algorithms automated the segmentation of nuclei and neurons and performed structure-specific feature extraction^36^.

Image preprocessing for the segmentation of nuclei was computed by convolving the raw Hoechst channel with a Gaussian filter. For the segmentation of dopaminergic neurons, a median filter was applied to the raw TH channel to generate a TH mask. TH-positive neuron fragmentation was measured using structuring elements for surface detection and was defined as the surface of the TH/TH mask. GFAP, S100β, and Tuj1 segmentation were performed via median filtering. The expression levels of these markers were expressed as (i) the sum of the positive pixels of the marker, and (ii) the percentage of cells positive for the marker among the total number of cells. For the latter, nuclei were segmented and a watershed function was applied. Considering the high cell density of the specimens, steps were taken to ensure a high-quality segmentation process, and nuclei with sizes greater than 10,000 pixels were removed. In the nuclei that were successfully segmented as a single element, a perinuclear zone was identified. If the marker of interest was positive in at least 1% of the perinuclear area, the cell was considered positive.

### Statistical analysis

The Kruskal–Wallis test was used for multiple comparisons in the quantitative analysis of astrocyte-related proteins, using JMP software. The Mann–Whitney test was performed when two conditions were compared in the image analysis of human MOs, using Matlab software.

### Reporting summary

Further information on research design is available in the Nature Research Reporting Summary linked to this article.

## Supplementary information

Reporting Summary Checklist

## Data Availability

The data that support the findings of this study are available from the corresponding authors upon reasonable request.
