# Supplementary material for: Reduced astrocytic reactivity in human brains and midbrain organoids with PRKN mutations
Source: NPJ Parkinsons Dis. 2020 Nov 13;6:33. doi: 10.1038/s41531-020-00137-8 (PMC7666226; doi:10.1038/s41531-020-00137-8)
Supplement: Supplementary file 1 — Reporting Summary Checklist [file 41531_2020_137_MOESM1_ESM.pdf]

nature research

Corresponding author(s): Masashi Takanashi, Nobutaka Hattori

Last updated by author(s): 2020/08/17

Reporting Summary

Nature Research wishes to improve the reproducibility of the work that we publish. This form provides structure for consistency and transparency in reporting. For further information on Nature Research policies, see our Editorial Policies and the Editorial Policy Checklist.

Statistics

For all statistical analyses, confirm that the following items are present in the figure legend, table legend, main text, or Methods section.

n/a	Confirmed	
☐	☒	The exact sample size (n) for each experimental group/condition, given as a discrete number and unit of measurement
☐	☒	A statement on whether measurements were taken from distinct samples or whether the same sample was measured repeatedly
☐	☒	The statistical test(s) used AND whether they are one- or two-sided<br>*Only common tests should be described solely by name; describe more complex techniques in the Methods section.*
☐	☒	A description of all covariates tested
☐	☒	A description of any assumptions or corrections, such as tests of normality and adjustment for multiple comparisons
☐	☒	A full description of the statistical parameters including central tendency (e.g. means) or other basic estimates (e.g. regression coefficient) AND variation (e.g. standard deviation) or associated estimates of uncertainty (e.g. confidence intervals)
☐	☐	For null hypothesis testing, the test statistic (e.g. F, t, r) with confidence intervals, effect sizes, degrees of freedom and P value noted<br>*Give P values as exact values whenever suitable.*
☐	☒	For Bayesian analysis, information on the choice of priors and Markov chain Monte Carlo settings
☐	☒	For hierarchical and complex designs, identification of the appropriate level for tests and full reporting of outcomes
☐	☒	Estimates of effect sizes (e.g. Cohen's d, Pearson's r), indicating how they were calculated

Our web collection on statistics for biologists contains articles on many of the points above.

Software and code

Policy information about availability of computer code

Data collection	No software was used.
Data analysis	JMP software (version 11.0.0, Cary, NC, USA), Matlab (v2017b, MathWorks, Natick, MA, USA)

For manuscripts utilizing custom algorithms or software that are central to the research but not yet described in published literature, software must be made available to editors and reviewers. We strongly encourage code deposition in a community repository (e.g. GitHub). See the Nature Research guidelines for submitting code & software for further information.

Data

Policy information about availability of data

All manuscripts must include a data availability statement. This statement should provide the following information, where applicable:

- Accession codes, unique identifiers, or web links for publicly available datasets
- A list of figures that have associated raw data
- A description of any restrictions on data availability

The data that support the findings of this study are available from the corresponding authors upon reasonable request.

Field-specific reporting

Please select the one below that is the best fit for your research. If you are not sure, read the appropriate sections before making your selection.

☒ Life sciences ☐ Behavioural & social sciences ☐ Ecological, evolutionary & environmental sciences

For a reference copy of the document with all sections, see nature.com/documents/nr-reporting-summary-flat.pdf

Life sciences study design

All studies must disclose on these points even when the disclosure is negative.

Sample size	Sample size were determined by the numbers we could get, because our samples were human autopsied brains.
Data exclusions	No data were excluded from the analyses.
Replication	We confirm all attempts at replication were successful.
Randomization	Randomization is not relevant to our study because our samples were limited human autopsied brains.
Blinding	Blinding is not relevant to our study because our samples were limited human autopsied brains.

Reporting for specific materials, systems and methods

We require information from authors about some types of materials, experimental systems and methods used in many studies. Here, indicate whether each material, system or method listed is relevant to your study. If you are not sure if a list item applies to your research, read the appropriate section before selecting a response.

Materials & experimental systems

n/a	Involved in the study	
☐	☒	Antibodies
☒	☐	Eukaryotic cell lines
☒	☐	Palaeontology and archaeology
☒	☐	Animals and other organisms
☐	☒	Human research participants
☒	☐	Clinical data
☒	☐	Dual use research of concern

Methods

n/a	Involved in the study	
☒	☐	ChIP-seq
☒	☐	Flow cytometry
☒	☐	MRI-based neuroimaging

Antibodies

Antibodies used

Anti-phosphorylated α-synuclein mouse monoclonal antibody, CAT# 015-29151, FUJIFILM Wako, Chuo-Ku, Osaka; clone psyn#64
Anti-TH mouse monoclonal antibody, CAT# T2928m, Sigma-Aldrich, St. Louis, MO, USA
Anti-nestin rabbit polyclonal antibody, CAT#18741, Imuno-Biological Laboratories (IBL), Fujioka, Gunma, Japan
Anti-GFAP mouse monoclonal antibody, CAT#11051, IBL, Fujioka, Gunma, Japan
Anti-vimentin mouse monoclonal antibody, CAT# GA63061-2J, Dako, Santa Clara, CA, USA
Anti-ALDH1L1 rabbit polyclonal antibody, CAT# 17390-1-AP, Proteintech, Rosemont, IL, USA
Anti-GFAP mouse monoclonal antibody, CAT# G3893, Sigma-Aldrich, St. Louis, MO, USA
Anti-vimentin mouse monoclonal antibody, CAT# V6630, Sigma-Aldrich
Anti-parkin mouse monoclonal antibody, CAT# 4211, Cell Signaling Technology, Danvers, MA, USA; clone PRK8
Anti-actin mouse monoclonal antibody, CAT# MAB1501, Millipore, Burlington, MA, USA; clone C4
Anti-GFAP chicken polyclonal antibody, CAT#AB5541, Millipore, Burlington, MA, USA
Anti-S100β mouse monoclonal antibody, CAT# AMAB91038, Sigma-Aldrich
Anti-tyrosine hydroxylase (TH) rabbit polyclonal antibody, CAT# 657012, Santa Cruz Biotechnology, Dallas, TX, USA

Validation

Anti-phosphorylated α-synuclein mouse monoclonal antibody, CAT# 015-29151: https://labchem-wako.fujifilm.com/us/product/detail/W01W0101-2519.html
Anti-TH mouse monoclonal antibody, CAT# T2928m: https://www.sigmaaldrich.com/catalog/product/sigma/T2928?lang=en&region=CA
Anti-nestin rabbit polyclonal antibody, CAT#18741: https://www.ibl-america.com/nestin-n1602-anti-human-rabbit-igg-affinity-purify/
Anti-GFAP mouse monoclonal antibody, CAT#11051: https://www.ibl-japan.co.jp/en/search/product/detail/id=3608
Anti-vimentin mouse monoclonal antibody, CAT# GA63061-2J: https://www.agilent.com/en-us/PageUnavailable?s=//www.agilent.com/en/product/immunohistochemistry/antibodies-controls/primary-antibodies/vimentin-(dako-omnis)-76243
Anti-ALDH1L1 rabbit polyclonal antibody, CAT# 17390-1-AP: https://www.ptglab.com/products/ALDH1L1-Antibody-17390-1-AP.htm
Anti-GFAP mouse monoclonal antibody, CAT# G3893: https://www.sigmaaldrich.com/catalog/product/sigma/g3893?lang=en&region=CA

Anti-vimentin mouse monoclonal antibody, CAT# V6630: https://www.sigmaaldrich.com/catalog/product/sigma/V6630?lang=en&region=CA
Anti-parkin mouse monoclonal antibody, CAT# 4211: https://www.cellsignal.jp/products/primary-antibodies/parkin-prk8-mouse-mab/4211
Anti-actin mouse monoclonal antibody, CAT# MAB1501: https://www.emdmillipore.com/US/en/product/Anti-Actin-Antibody-clone-C4,MM_NF-MAB1501?bd=1
Anti-GFAP chicken polyclonal antibody, CAT#AB5541: https://www.merckmillipore.com/JP/en/product/Anti-Glial-Fibrillary-Acidic-Protein-Antibody,MM_NF-AB5541
Anti-S100β mouse monoclonal antibody, CAT# AMAB91038: https://www.sigmaaldrich.com/catalog/product/sigma/amab91038?lang=en&region=CA
Anti-tyrosine hydroxylase (TH) rabbit polyclonal antibody, CAT# 657012: https://www.merckmillipore.com/JP/en/product/Anti-Tyrosine-Hydroxylase-Rabbit-pAb,EMD_BIO-657012?ReferrerURL=https%3A%2F%2Fwww.google.com%2F

Human research participants

Policy information about studies involving human research participants

Population characteristics	All participants were Japanese from 50 y/o to 81y/o including 13 males and 4 females.
Recruitment	All participants were accepted autopsy and postmortem research by their families.
Ethics oversight	The ethics committee of the Juntendo University School of Medicine

Note that full information on the approval of the study protocol must also be provided in the manuscript.
